# Sequential Bilateral Cochlear Implantation via the Transmastoid Labyrinthotomy Approach Using Custom‐Made Electrodes in a Pediatric Patient With Common Cavity Deformities: A Case Report

**DOI:** 10.1155/crot/9913363

**Published:** 2026-08-05

**Authors:** Ciro Lucio Vigliaroli, Millo Achille Beltrame, Lazzaro Cassano, Francesca Pia Cavalluzzo, Deniis Curatolo, Alessandra Rossato, Michele Cassano, Eleonora M. C. Trecca

**Affiliations:** ^1^ Department of Otorhinolaryngology and Maxillofacial Surgery, Hospital Casa Sollievo della Sofferenza, San Giovanni Rotondo, Foggia, Italy; ^2^ Department of Otorhinolaryngology, Santa Maria del Carmine Hospital, Rovereto, Trento, Italy; ^3^ Department of Clinical and Experimental Medicine, Unit of Otorhinolaryngology-Head and Neck Surgery, University of Foggia, Foggia, Italy, unifg.it

**Keywords:** cochlear implants, common cavity, electrodes, hearing loss, inner ear malformations, labyrinthotomy

## Abstract

Inner ear malformations (IEMs) present significant challenges for cochlear implantation (CI), particularly the common cavity deformity, in which the cochlea and vestibule merge into a single space. Surgical and audiological outcomes in such cases depend on accurate electrode placement, nerve integrity, and tailored surgical strategies. We report the case of a pediatric patient with bilateral common cavity deformities who underwent sequential bilateral CI using the double posterior transmastoid labyrinthotomy (TML) approach with custom‐made electrodes. The patient, diagnosed with profound bilateral sensorineural hearing loss at 1 year of age, showed bilateral common cavities with identifiable but reduced caliber seventh and eighth cranial nerves on radiological imaging and absent responses on auditory brainstem response testing. Based on preoperative imaging, a custom‐made MED‐EL FORM19 electrode (Innsbruck, Austria) was selected and adapted to match the patient’s anatomy, aiming to optimize placement and reduce intraoperative complications. A staged implantation was performed. Surgery was uneventful, with intraoperative neural response telemetry confirming responses in both ears. At four‐year follow‐up, aided thresholds were stable in the speech frequency range (right ear: 40–60 dB HL; left ear: 50–65 dB HL). Speech audiometry in quiet demonstrated good detection and recognition, and language development was appropriate for hearing age, with significant improvement from baseline. This case illustrates that double posterior TML with custom‐made electrodes is a safe and effective strategy for CI in complex IEMs such as common cavity deformities. The technique offers precise electrode placement, minimizes risks to the facial nerve, and ensures stable fixation. Early bilateral stimulation, active family involvement, multidisciplinary care in specialized centers, and tailored electrode design are critical to achieving favorable long‐term outcomes. Multicenter studies are needed to further validate these findings and refine patient selection.

## 1. Introduction

Inner ear malformations (IEMs) are complex congenital anomalies that can manifest in various phenotypes, affecting the cochlea, vestibule, or both. They exhibit a wide range of characteristics, including variations in the facial nerve’s course and the potential for intraoperative complications, such as cerebrospinal fluid leakage. Audiological manifestations can span from mild to profound sensorineural hearing loss (SNHL) and may affect one or both ears. Treatment options for IEMs typically involve hearing aids, cochlear implants (CIs), and auditory brainstem implants (ABIs). IEMs continue to present a formidable challenge for ear surgeons and audiologists, particularly when deciding on the most appropriate treatment for a specific IEM subtype and SNHL pattern [1].

One rare form of IEM is the “common cavity” deformity, where the cochlea and vestibule merge into a single cavity rather than forming distinct structures. The prevalence of IEMs among pediatric CI candidates has been estimated at approximately 20%, with the common cavity representing a rare subset, accounting for approximately 1% of all IEMs in most large series, although reported frequencies may vary according to classification systems, referral patterns, and study inclusion criteria [2]. The feasibility of CIs in individuals with this common cavity deformity hinges on several factors, including the precise anatomical characteristics of the malformation and the condition of the vestibulocochlear nerve. While it is no longer regarded as an absolute contraindication to CI, outcomes remain less predictable than in anatomically normal cochleae, making optimal electrode placement and nerve integrity crucial for auditory benefit [3].

Various surgical techniques have been described to address this issue, including the traditional transmastoid approach, a canal‐wall down mastoidectomy with external auditory canal closure, and, more recently, endoscopic‐guided electrode placement [4]. As an alternative aimed at minimizing the risk of complications, a transmastoid labyrinthotomy (TML) approach has also been reported in the literature, offering a safe route with respect to the facial nerve, facilitating management of potential intraoperative cerebrospinal fluid gushers, and enabling secure stabilization of the electrode within the cavity [5].

Regarding the electrode choice in cases of complex IEMs, the use of custom‐made electrodes can offer significant surgical and audiological advantages. These electrodes are specifically designed to accommodate the patient’s unique cochlear or vestibular anatomy, allowing optimal positioning within the malformed cavity. Their tailored length, flexibility, and tip design reduce the risk of trauma to neural structures, minimize misplacement, and facilitate stable fixation. In common cavity deformities, custom‐made arrays can improve the likelihood of stimulating the residual cochlear nerve fibers while reducing the probability of postoperative migration, thereby enhancing long‐term auditory performance [6, 7].

The purpose of this report is to provide a step‐by‐step description of sequential bilateral CI using the double posterior TML approach in a pediatric patient with bilateral common cavity deformities and to discuss the audiological outcomes achieved with custom‐made electrodes.

## 2. Case Presentation

This report was developed according to the Surgical CAse REport (SCARE) guidelines [8], and parental informed consent was obtained.

**FIGURE 1:**
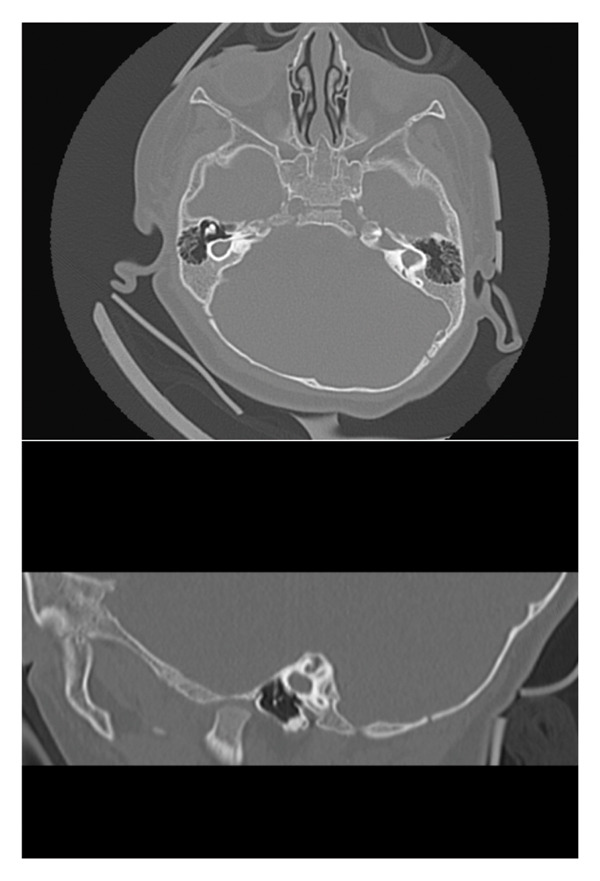
High‐resolution computed tomography (CT) demonstrating bilateral common cavity deformities. The selected axial and coronal reconstructions were chosen to allow simultaneous visualization of the malformation on both sides and show the absence of normal cochlear partitioning, with a single cystic cavity representing fused cochlear and vestibular structures, consistent with common cavity deformity.

**FIGURE 2:**
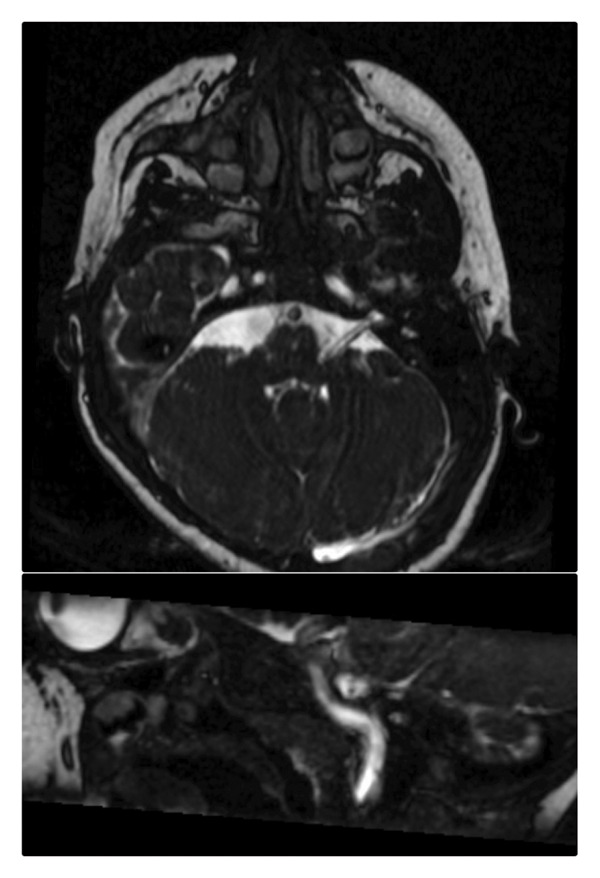
High‐resolution magnetic resonance imaging (MRI) of the temporal bones demonstrating the abnormal inner ear anatomy associated with bilateral common cavity deformities. Both superior semicircular canals and the left posterior semicircular canal are identifiable. MRI was primarily used to assess the vestibulocochlear nerves, which were bilaterally identifiable although reduced in caliber.

A 1‐year‐old male infant with suspected SNHL was referred to our audiology clinic for further evaluation. Auditory brainstem response (ABR) testing revealed no detectable responses at maximum stimulation levels (≥ 95 dB nHL). Computed tomography (CT) revealed bilateral common cavity deformities involving both the vestibule and cochlea (Figure 1). Magnetic resonance imaging (MRI) confirmed these findings and demonstrated bilaterally identifiable seventh and eighth cranial nerves with reduced caliber. Regarding the semicircular canals, both superior canals and the left posterior canal were identifiable, while the lateral canals were absent (Figure 2).

**FIGURE 3:**
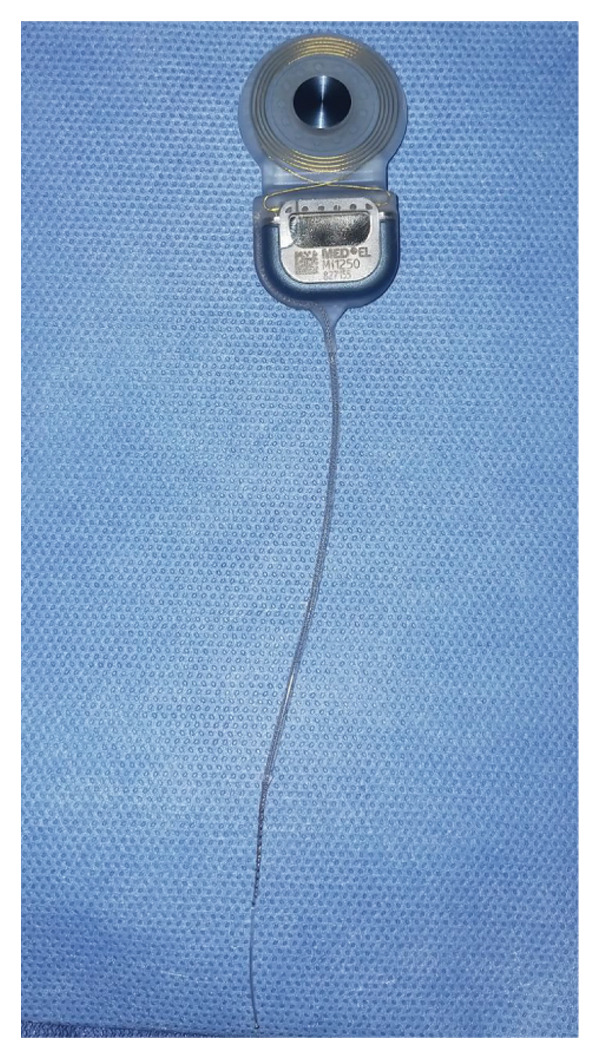
Custom‐made MED‐EL FORM19 electrode designed for common cavity deformities (MED‐EL, Innsbruck, Austria). The array incorporates 12 active contacts and a 20‐mm passive apical silicone extension terminating in a platinum ball, intended to facilitate atraumatic insertion, stable positioning within the common cavity, and proximity of the stimulating contacts to residual neural tissue.

Following multidisciplinary evaluation and family counseling, particular attention was devoted to the assessment of the vestibulocochlear nerves. Although bilaterally reduced in caliber, the vestibulocochlear nerves were clearly identifiable on MRI and were therefore considered potentially functional. Although no additional preoperative electrophysiological tests beyond conventional ABR specifically assessing cochlear nerve function were performed, this finding represented the principal factor supporting CI candidacy and favoring CI over ABI as the initial rehabilitative strategy, whereas ABI was reserved as a possible alternative should auditory benefit prove insufficient after the first CI. The decision for a staged approach was based on optimizing auditory rehabilitation and minimizing surgical risk management. Based on the preoperative radiological assessment, a custom‐made MED‐EL FORM19 electrode (MED‐EL, Innsbruck, Austria) was selected and adapted to the patient’s specific inner ear anatomy (Figure 3). The array consisted of 12 active contacts and featured a 20‐mm atraumatic apical silicone extension ending in a platinum ball. Compared with conventional CI electrode arrays, this design was specifically developed for common cavity deformities, allowing the passive silicone extension to be advanced within the malformed cavity while facilitating stable positioning of the stimulating contacts along the cavity wall. This configuration was intended to maximize contact with residual neural tissue, improve electrode stability, and reduce the risk of migration.

The left ear was implanted in March 2021 at age the of 1 year 6 months, and the right ear in September 2022 at the age of 2 years.

### 2.1. Surgical Technique

In this surgical procedure, a standard incision was made and extended approximately 8 cm posteriorly. The skin and periosteal flap were elevated, and the implant site was prepared. A bone island was fashioned when the temporal squama was thin to provide additional stability.

Mastoidectomy was then initiated, progressing toward the aditus ad antrum under microscopic visualization until enchondral bone of the otic capsule was exposed.

Two labyrinthotomies were performed, with the endosteum opened. The superior labyrinthotomy was placed near the nonampullated end of the lateral semicircular canal, and the inferior one approximately 4 mm below. The posteriorly directed drilling trajectory was specifically selected to maintain a safe distance from the facial nerve and to maximize exposure of the common cavity while preserving surrounding neurovascular structures. Continuous facial nerve monitoring was employed throughout the procedure using standard electromyographic monitoring systems.

**FIGURE 4:**
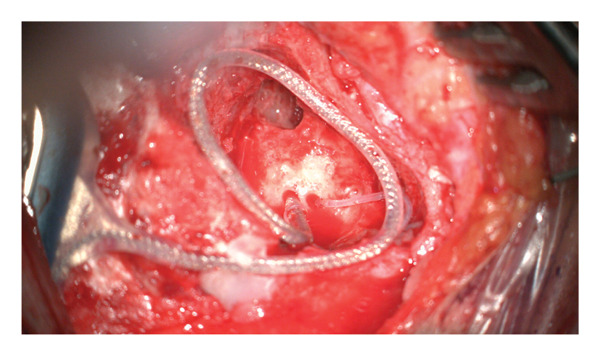
Intraoperative view of the right ear showing the double posterior labyrinthotomy technique. The superior and inferior labyrinthotomy openings are visible after insertion of the custom‐made electrode into the common cavity.

The electrode was prepared for insertion, with its nonactive portion ending in a small ball. This nonactive segment was advanced through the superior labyrinthotomy, visualized via the inferior one, and positioned within the common cavity (Figure 4). No neuronavigation or image‐guided surgical system was required. Electrode placement was performed under microscopic visualization using anatomical landmarks. The temporalis fascia was used to seal the labyrinthotomy sites, and fibrin glue may be applied. If required, bone pâté obliteration can be performed. The connecting wires and receiver/stimulator were anchored to the mastoid and temporal squama, and the wound was closed in layers.

**FIGURE 5:**
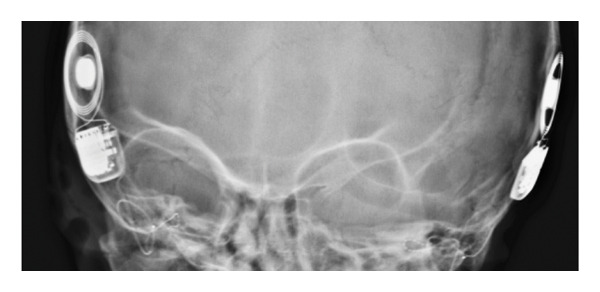
Postoperative X‐ray confirming the correct placement of both cochlear implants (CIs).

No intraoperative complications, such as cerebrospinal fluid leakage, occurred. Intraoperative neural response telemetry demonstrated present responses in both ears. Electrode position was verified intraoperatively and subsequently confirmed by postoperative radiography (Figure 5).

The patient recovered uneventfully and was discharged in good condition after a 2‐day hospital stay, with standard postoperative care instructions provided to the family.

### 2.2. Audiological Outcomes

At the 4‐year follow‐up after sequential bilateral CI, audiological assessment demonstrated stable aided thresholds in the speech frequency range for both ears. Pure‐tone audiometry revealed hearing thresholds ranging from 40 to 60 dB HL across tested frequencies in the right ear and from 50 to 65 dB HL in the left ear. Speech audiometry, performed in quiet using bisyllabic word lists, showed good speech detection and recognition abilities, consistent with functional auditory benefit from the bilateral implants. Language development was appropriate for hearing age, with significant improvement noted compared to preimplant baseline. No device‐related complications, electrode migration, or deterioration of auditory performance in thresholds or speech perception was observed during follow‐up.

## 3. Discussion

To the best of our knowledge, only one other report in the literature has described bilateral CI in a pediatric patient with a common cavity malformation, using a TML approach and a standard electrode [9]. The present case adds to the limited literature by demonstrating that a double posterior TML approach combined with a custom‐made electrode can provide a safe, anatomically adapted, and functionally effective solution for this challenging malformation.

Auditory outcomes in common cavity deformities remain inherently variable because of the unpredictable distribution of residual neural tissue along the cavity wall and the heterogeneous degree of cochlear nerve development. The use of a customized electrode is supported by multicenter data indicating that children with common cavity implanted with such electrodes show steady and significant audiological improvement, including gains in Categories of Auditory Performance (CAP) and Speech Intelligibility Rating (SIR) over time. Although custom electrodes did not necessarily reduce electrode migration or surgical complications according to Zhang et al., they remain foundational for achieving precise placement and long‐term functional stability [10]. Moreover, custom‐designed arrays have been shown to potentially optimize outcomes in children with IEMs [11].

In our case, the double posterior TML approach—positioned posteriorly relative to the facial nerve—helped mitigate the risk of nerve injury and manage intraoperative CSF gushers effectively. Additionally, electrode positioning within the cavity was stable, which likely contributed to consistent audiological mapping and minimal adjustments postoperatively. Unlike conventional CI arrays designed for a normally partitioned cochlea, a custom‐made FORM19 electrode incorporating 12 active contacts and a 20‐mm passive silicone apical extension was specifically intended for implantation within a common cavity. The silicone extension terminating in a platinum ball allowed controlled advancement within the cavity while maintaining the stimulating contacts in close proximity to the cavity wall, where residual neural elements are expected to be concentrated. This design may contribute to improved electrode stability, reduced migration risk, and more consistent electrical stimulation over time. In our patient, no electrode migration or device‐related complications were observed during the 4‐year follow‐up period.

Early bilateral stimulation is crucial for auditory and language development. Evidence supports this, showing that children with bilateral implants outperform their unilaterally implanted peers in speech perception, sound localization, and receptive vocabulary [12]. Coupled with active family involvement, with comprehensive preoperative counseling and ongoing rehabilitation, this approach significantly enhances educational integration and long‐term communication outcomes.

Bilateral CI is widely recognized as offering superior auditory and linguistic outcomes, such as improved speech perception in quiet and noise, sound localization, and speed of language acquisition, compared to unilateral CI or ABI, particularly in pediatric populations with sufficient cochlear nerve function [13, 14].

The ABI, which bypasses the cochlea and cochlear nerve to stimulate the cochlear nucleus, remains an alternative in cases where CI is not feasible, such as cochlear aplasia, absent auditory nerve, or severely ossified cochlea. However, outcomes with ABI tend to be more modest: Whereas CI often supports language development and open‐set recognition, ABI users commonly achieve primarily environmental sound awareness and basic speech awareness, often requiring lip‐reading support.

In patients with reduced‐caliber cochlear nerves, such as in our case, it is critical to conduct multidimensional candidacy assessments. Clinical guidelines and recent surveys suggest that CI should generally be considered the first‐line rehabilitative option whenever a recognizable cochlear nerve is present, reserving ABI for cases of cochlear nerve aplasia or unsatisfactory progress following implantation. Although no additional preoperative electrophysiological tests beyond conventional ABR specifically assessing cochlear nerve functionality were performed in our patient, high‐resolution MRI demonstrated bilaterally identifiable vestibulocochlear nerves despite their reduced caliber [15].

Furthermore, measurable intraoperative neural response telemetry was obtained in both ears, providing additional functional evidence of an electrically responsive auditory pathway. Together, these findings supported the feasibility of CI despite the severity of the malformation. The favorable long‐term audiological outcomes observed in our patient further confirm the appropriateness of the initial CI approach, even in the presence of severe IEMs and cochlear nerve hypoplasia.

Surgical success in such complex cases relies heavily on multidisciplinary management in specialized centers capable of customizing electrodes and adapting surgical techniques to the child’s anatomy. Long‐term outcomes will benefit from consistent follow‐up, audiologic monitoring, and speech therapy integrated with school support.

The main limitation of this study is its nature as a single case report, which inherently limits the generalizability of the findings. Further studies involving larger case series or comparative analyses are needed to determine the optimal surgical approach and electrode choice for patients with common cavity malformation. In addition, fitting and mapping may be challenging in common cavity patients because of atypical current spread and the potential occurrence of nonauditory stimulation. More evidence is required to define the most effective strategies in this specific population. Despite encouraging outcomes, auditory performance in common cavity deformities remains highly variable and depends on multiple factors including neural integrity, cavity morphology, electrode positioning, rehabilitation intensity, and surgical expertise. Although the present patient demonstrated stable auditory performance during follow‐up, longer‐term data extending into adolescence and adulthood are still required to confirm the durability of these outcomes. Finally, potential variability in surgical expertise and intraoperative decision‐making should be considered when interpreting the applicability of this technique in different clinical settings.

## 4. Conclusions

Bilateral CI using a double posterior TML approach with custom‐made electrodes is a feasible and effective intervention for pediatric patients with bilateral common cavity deformities. This strategy enables precise electrode placement and stable fixation, tailored to individual anatomy, and enhanced bilateral auditory stimulation, which is indispensable for speech, language, and binaural hearing development. Nevertheless, multicenter prospective studies with larger cohorts are needed to validate these findings, refine patient selection criteria, and assess long‐term auditory, linguistic, and functional outcomes across complex IEM subtypes.

## Funding

No funding was received for this manuscript. Open access publishing facilitated by Universita degli Studi di Foggia, as part of the Wiley ‐ CRUI‐CARE agreement.

## Consent

Patient consent was obtained.

## Conflicts of Interest

The authors declare no conflicts of interest.

## Data Availability

The data that support the findings of this study are available from the corresponding author upon reasonable request.
